# Efficacy of COVID-HIGIV in animal models of SARS-CoV-2 infection

**DOI:** 10.1038/s41598-022-21223-2

**Published:** 2022-10-10

**Authors:** Aruni Jha, Douglas Barker, Jocelyne Lew, Vinoth Manoharan, Jill van Kessel, Robert Haupt, Derek Toth, Matthew Frieman, Darryl Falzarano, Shantha Kodihalli

**Affiliations:** 1 Research and Development, Emergent BioSolutions, Winnipeg, MB Canada; 2 Vaccine and Infectious Disease Organization (VIDO), University of Saskatchewan, Saskatoon, SK Canada; 3 Department of Veterinary Microbiology, University of Saskatchewan, Saskatoon, SK Canada; 4 Department of Microbiology and Immunology, University of Maryland School of Medicine, Baltimore, MD USA

**Keywords:** Viral infection, SARS-CoV-2

## Abstract

In late 2019 the severe acute respiratory syndrome coronavirus 2 (SARS-CoV-2) virus emerged in China and quickly spread into a worldwide pandemic. It has caused millions of hospitalizations and deaths, despite the use of COVID-19 vaccines. Convalescent plasma and monoclonal antibodies emerged as major therapeutic options for treatment of COVID-19. We have developed an anti-SARS-CoV-2 immunoglobulin intravenous (Human) (COVID-HIGIV), a potential improvement from using convalescent plasma. In this report the efficacy of COVID-HIGIV was evaluated in hamster and mouse models of SARS-CoV-2 infection. COVID-HIGIV treatment in both mice and hamsters significantly reduced the viral load in the lungs. Among COVID-HIGIV treated animals, infection-related body weight loss was reduced and the animals regained their baseline body weight faster than the PBS controls. In hamsters, COVID-HIGIV treatment reduced infection-associated lung pathology including lung inflammation, and pneumocyte hypertrophy in the lungs. These results support ongoing trials for outpatient treatment with COVID-HIGIV for safety and efficacy evaluation (NCT04910269, NCT04546581).

## Introduction

In 2019 a novel coronavirus designated as SARS-CoV-2 emerged in Wuhan, China, causing pneumonia and respiratory failure termed Coronavirus Disease 2019 (COVID-19)^1,2^. The SARS-CoV-2 has resulted in more than 600 million confirmed cases and over six million deaths worldwide as of April 6, 2022^3^. The scale of the pandemic has posed an extraordinary threat to global public health^4,5^. To counter the COVID-19 pandemic, two vaccines have been approved by US Food and Drug Administration (FDA), while others are used under Emergency Use Authorization (EUA)^6^. Although vaccines are effective^7^, herd immunity is far from being achieved in most countries. Several therapeutics have also been tested in the clinic; however, only remdesivir has been approved for treatment of COVID-19^8^. Other antivirals such as PAXLOVID^9,10^ and LEGEVRIO^11,12^ are available under EUA. Dexamethasone has also been recommended for use in hospitalized COVID-19 patients^13^.

Passive immunizations with monoclonal (mAb) and polyclonal antibodies (pAb) have emerged as promising strategies for treating emerging infectious diseases. The Food and Drug Administration (FDA) has granted EUA to several mAb therapies^14–17^ for patients with mild to moderate COVID-19 and patients at high risk of progressing to severe COVID-19, and several others in clinical trials^18^. Despite the promising data, mAbs have limitations, including cost, the inability to provide broad cross-reactive protection and the concern about variants containing Spike mutations that make them ineffective^19^. In fact, FDA revised the EUA for mAbs bamlanivimab and etesevimab (administered as a cocktail) and REGEN-COV to limit their use because they are not active against the omicron variant, which is circulating at a high frequency in the US^20^.

Convalescent plasma has previously demonstrated clinical benefits against both viral and bacterial infections^21–24^. The efficacy of convalescent plasma in treating severe respiratory illnesses caused by the original SARS-CoV infection suggested clear clinical benefits, including better survival, viral load reduction and earlier discharge from the hospital^25–27^. Similarly, published reports demonstrated the benefit of treatment of COVID-19 patients with convalescent plasma, including resolution of fever, reduction in viral load, alleviation of respiratory symptoms, improvements in supplemental oxygen requirements and enhanced survival compared to controls^28–30^. However, a recent meta-analysis report suggests that convalescent plasma is not associated with a clinical benefit^31^. FDA has limited EUA for convalescent plasma to patients with an immunosuppressive disease or receiving immunosuppressive treatments^32^. Currently the WHO recommends against the use of convalescent plasma except in clinical trials for severe and critical COVID-19 patients^33^.

One of the common drawbacks of convalescent plasma is the high donor-to-donor variability in neutralization titers^34^, however, in a product such as COVID-HIGIV this limitation is overcome by the purification and qualification process. COVID-HIGIV is a human hyperimmune product manufactured from donor plasma enriched for anti-SARS-CoV-2 binding and neutralizing activity. Here, the therapeutic potential of COVID-HIGIV was evaluated in animal models of SARS-CoV-2 infection to support ongoing clinical development of COVID-HIGIV.

For our studies, a mouse model (mouse transduced with adenovirus carrying human angiotensin-converting enzyme-2 (ACE-2)) and hamster model was used for COVID-HIGIV efficacy evaluation^35,36^. Syrian hamsters have been used extensively to study SARS-CoV-2 infection, with SARS-CoV-2 replicating efficiently, demonstrating clinical disease with rapid weight loss, very high lung viral load and severe lung pathology. Thus, this model may more closely mimic more severe disease in humans^37–40^. Since wild type mice are not susceptible to SARS-CoV-2 infection as the SARS-CoV-2 spike protein lacks affinity to mouse ACE2, required for the entry of the virus^41,42^, a variety of mouse models have been developed where the mice express the human ACE2 (hACE2) via genetic manipulation^43–45^ or viral transduction^35^. Mouse-adapted SARS-CoV-2 viral strains have also been developed to study infection in wild type mice^46,47^. While transgenic mice are susceptible to SARS-CoV-2 and provide a severe disease model that recapitulates features of human disease including infection-related mortality, a major drawback is the non-physiological expression of the hACE2, which is independent of the complex regulatory system that controls the expression of ACE2. Moreover, it is not completely understood if lethality is primarily due to severe lung infection or viral encephalitis^48,49^. Mouse adapted SARS-CoV-2 viruses provide an easier model to study SARS-CoV-2 infection, where wild type mice (non-susceptible to SARS-CoV-2 infection) are used that alleviates logistical challenges associated with other mouse models^46,47^. Transduction with adenovirus or adeno-associated virus expressing hACE2 (Ad5-hACE2 or AAV-hACE2, respectively) results in mice susceptible to infection by sensitizing the respiratory tract^35^. This permits the transient replication of SARS-CoV-2 in the lungs of mice for several days, which leads to development of clinical disease characterized by efficient replication of SARS-CoV-2 in lungs, body weight loss and lung pathology^35,49–51^.

The effectiveness of COVID-HIGIV on clinical morbidity and viral burden was measured in both mice with adenovirus expressing human ACE2 and hamster models of SARS-CoV-2 infection. The efficacy of COVID-HIGIV on SARS-CoV-2 induced lung pathology in hamsters was also assessed. Findings from these studies demonstrate that COVID-HIGIV is efficacious in multiple relevant models of COVID-19, where it reduced viral load, tissue damage and inflammation in the lungs in a dose-dependent manner.

## Materials and methods

### Ethics

This research was conducted at the University of Maryland School of Medicine (Baltimore, MD) and the Vaccine and Infectious Disease Organization (University of Saskatchewan; Saskatoon, Canada). Before the commencement of animal work, prior approval from the respective Institutional Animal Care and Use Committee (IACUC) at the University of Maryland and the University of Saskatchewan was obtained. All animal work was carried out according to the Animal Welfare Act (AWA 7 USC §2131 2002, 2007 and 2008), guidelines set by the Canadian Council on Animal Care (CCAC) and ARRIVE guidelines set by the National Centre for the Replacement Refinement and Reduction of Animals in Research (NC3Rs). With regard to the human plasma used in the manufacture of COVID-HIGIV, informed consent from plasma donors was obtained by the plasma centers prior to initiation of plasma collection activities.

### Viruses

All work with infectious virus was conducted in BSL-3 (biosafety level 3) facilities. SARS-CoV-2 strain WA-1 (NR-52281, GISAID:EPI-ISL-404895) was received from BEI resources and expanded in Vero E6 cells^52^. The titer of the stock was determined by plaque assay using Vero E6 cells as described previously^53^. The SARS-CoV-2/Canada/ON/VIDO-01/2020/Vero’76/p.2 (GISAID:EPI-ISL-45177) used in these studies was isolated from a clinical specimen obtained at the Sunnybrook Research Institute (SRI)/University of Toronto on VeroE6 cells and provided by the Vaccine and Infectious Disease Organization (VIDO). Following isolation, the virus was expanded in Vero76 cells (ATCC CRL 1587) to generate a challenge virus stock. Both strains are essentially identical sharing 99.98% sequence identity over 99% of the genome. The titer of the stock was determined by TCID_50_ assay using Vero’76 cells as described previously^54^.

### COVID-HIGIV manufacture

COVID-HIGIV is a purified human IgG product manufactured by Emergent BioSolutions Canada Inc (Winnipeg, Canada) using plasma collected by commercial plasma collection companies from convalescent donors previously infected with SARS-CoV-2. The immunoglobulin fraction was purified using a scalable (200–1000 L plasma) established manufacturing process which includes anion-exchange chromatography and two orthogonal virus removal steps (filtration and solvent/detergent treatment). Three COVID-HIGIV lots with a total protein concentration of ~ 100 mg/mL (Pilot lot # PD_740_POC_17_001_006) (Clinical lots # 23003584 and 22002290) containing > 99% human IgG were used in this study. Neutralizing potency against wild-type SARS-CoV-2 for lots of COVID-HIGIV used for this work was 763 Alliance Units (AU)/mL (mouse study and in vitro variant testing, lot # PD_740_POC_17_001_006, total protein 100 mg/mL), 1089 AU/mL (hamster study, lot # 23003584, total protein 107 mg/mL) and 694.5 AU/mL (in vitro variant testing, lot#22002290, total protein 103 mg/mL).

### Neutralization assay

COVID-HIGIV potency was determined against the Alliance IgG standard (purified IgG from convalescent plasma). The Alliance standard has an arbitrarily assigned unitage of neutralizing potency (Alliance units; AU) and COVID-HIGIV potency is reported in AU/mL. Potency testing was performed at Integrated Research Facility/NIAID lab using a SARS-CoV-2 wild type neutralization assay^55^. This assay directly measures the neutralizing activity of pooled plasma and/or COVID-HIGIV product through the quantitation of infection of Vero cells by wild-type SARS-CoV-2 (WA1/2020; BEI Cat#NR52281). Briefly, samples were serially diluted through a six-step twofold serial dilution (1:40–1:1280) in serum free DMEM using a 96 well format. SARS-CoV-2 was diluted in serum free DMEM to multiplicity of infection (MOI) 0.5 (for example 15,000 PFU per 30,000 cells). The diluted samples were mixed with diluted virus 1:1 and incubated at 37 ℃ for 1 h. The virus-sample mixture was then added to each well of the 96 well plate containing Vero E6 cells and incubated under 5% CO_2_ at 37 ℃ for 24 h. Cells were fixed with formalin and probed with SARS-CoV/SARS-CoV-2 N protein specific rabbit mAb. Cells were then washed before re-probing with an Alexa Fluor 594-conjugated goat anti-rabbit IgG secondary antibody in the dark for 1 h at room temperature and counter stained with Hoechst nuclear stain. Using a high content imaging system number of SARS-CoV-2 positive cells were counted in four independent fields, with > 1000 cells/field. The number percent positive wells relative to untreated controls were determined and the mean of four replicates/dilution was plotted against the concentration of each dilution. A four-parameter logistical analysis was performed to determine 50% neutralization value^55^.

Live virus variant testing was also done at NIAID/IRF (under BSL-3) against the variants Alpha (hCoV-19/USA/CA_CDC_5574/2020; BEI Resources Cat# NR-54011), Beta (hCoV-19/South Africa/KRISP-K005325/2020; BEI Cat# NR-54009), Gamma (hCoV-19/Japan/Ty7-503/2021; BEI Cat# NR-54984), Delta (hCoV-19/USA/MD-HP05285/2021; BEI Cat #NR-55673) and Omicron (hCoV-19/USA/MD-HP20874/2021 B.1.1.529; BEI Cat #NR-56461)) using the same method as described above for determining neutralizing potency, but with wild type SARS-CoV-2 (WA1/2020) used as a control to determine fold reduction/increase in neutralization to each variant tested.

### Therapeutic evaluation of COVID-HIGIV in mice

#### Human ACE2 (hACE2) transduction in mice

Wild type (BALB/c) mice are not susceptible to SARS-CoV-2 infection^35,49,50^; however, transduction with adenovirus expressing human ACE2 (Ad5-hACE2) sensitizes the respiratory tract to SARS-CoV-2 infection, resulting in a self-limiting infection model characterized by transient body weight loss, viral replication in lung and associated lung pathology. Ninety-six female, specific pathogen free BALB/c mice 8–10 weeks of age were randomly assigned to cages upon receipt from Charles River Laboratories. Individual mice in each cage were identified by ear punches. Mice were anesthetized using ketamine-xylazine, and the respiratory tract of these animals was transduced via intranasal (i.n) inoculation with 2.5 × 10^8^ PFU of recombinant adenovirus (50 mL) prepared from Ad5/hACE2 stock (University of Iowa Viral Vector Core).

#### COVID-HIGIV evaluation in mice infected with SARS-CoV-2

Four days after adenovirus transduction, mice were anesthetized again and were divided into five groups of 18 each and challenged i.n with SARS-CoV-2 strain WA-1 (1 × 10^5^ PFU in 50 mL). The remaining six were sham infected for use as histopathological controls. Six hours after SARS-CoV-2 challenge animals were treated by intraperitoneal (i.p.) injection with either PBS or one of four COVID-HIGIV doses (6.25, 25, 100 and 400 mg/kg) in a final volume of 100 µL. Body weight of all animals in each group was measured daily until the sacrifice day or 14th day post-infection (the final day of the study). Six animals per group were sacrificed at 2- and 4-days post infection (dpi) and lungs harvested for SARS-CoV-2 viral load and histopathological analysis.

#### Viral load assessment assays for lungs from mice

Viral loads were measured in duplicate by plaque assay across a 6-point dilution curve as described previously^52^. Briefly, Vero cells were seeded in 35 mm dishes with 5 × 10^5^ cells/dish 24 h prior to infection. Supernatant from lung homogenates were serially diluted from 10^–1^ to 10^–6^ in serum free medium. Before infection cells were washed with the serum free medium and then inoculated with 200 µL lung homogenates at different dilutions for 1 h at 37 ℃. The inoculum was gently rocked every 10 min. After 1-h cells were washed with serum free medium and 1:1 mixture of DMEM (2x) and 1.6% agarose was added to each well, plates were incubated at 37 ℃ for 72 h later plaques were counted. Plaque assay was performed in a blinded manner.

### Evaluation of COVID-HIGIV in hamsters

#### COVID-HIGIV evaluation in hamsters infected with SARS-CoV-2

Wild type Syrian golden hamsters are sensitive to intranasal challenge with SARS-CoV-2. As is the case for adenovirus transduced mice, this model is characterized by transient body weight loss, viral replication in lung and associated lung pathology^39,56,57^. Seventy-two specific pathogen free golden Syrian hamsters 5–7 weeks of age were housed in cages upon receipt from Charles River Laboratories. Individual hamsters in each cage were identified by microchip and an ear notch. Animals were randomized into 4 groups (n = 18/group) and infected via i.n under anesthesia (3% isoflurane delivered in 100% oxygen by inhalation in an induction chamber) with 10^5^ TCID_50_ of SARS-CoV-2/Canada/ON/VIDO-01/2020 (1 × 10^5^ TCID_50_ in 100 mL) on Day 0. Six hours prior to infection, all animals were treated via i.p. with either PBS or one of three COVID-HIGIV doses (100, 400 or 800 mg/kg). Nine animals per treatment group were sacrificed on 3 dpi and lungs collected for viral load analysis. The remaining nine animals per treatment group were sacrificed on 10 dpi and lungs collected for histopathological analysis as described below. An additional two animals were sham infected and used as histopathological controls.

#### Viral load assessment by RT-qPCR in hamster lungs

Immediately after harvesting lung tissues at 3 dpi, a section of lung tissue was placed in RNAlater (Qiagen cat #1018087) at necropsy, stored at 4 °C for 2 days and then frozen at − 80 °C. Subsequently, extraction of RNA was done using lysis buffer (RLT Qiagen) with a 5 mm sterile stainless-steel bead in the TissueLyserII (Qiagen) for 6 min, at 30 Hz. The solution was centrifuged at 5000×*g* for 5 min. Supernatant was transferred to a fresh tube with additional RLT and the tube was incubated at room temperature for 10 min. The supernatant was then transferred to a new tube containing 600 µL of 70% ethanol. RNA was purified using Qiagen RNeasy Mini Kit (Cat No/ID: 74106) and eluted with 50 µL elution buffer.

The RT-qPCR assays for the sub-genomic (sg) RNA were performed using SARS-CoV-2 specific primers (Table 1) and Qiagen Quantifast RT-PCR Probe kits (Cat No/ID: 204454). For sgRNA, a standard curve of RNA detected by RT-qPCR was generated using serially diluted RNA isolated from a stock virus tittered at 2 × 10^6^ TCID_50_/ml. RNA was serially diluted (tenfold) to a final concentration range of 2 × 10^5^ to 20 copies per reaction. The Ct values for individual samples were used with a standard curve to determine the number of TCID_50_ equivalents/mL in each reaction.

**Table 1:** Sequence of primers.

Primer	Sequence
Forward primer (Fwd)—sgRNA	CGATCTCTTGTAGATCTGTTCTC
Reverse primer (Rev)	ATATTGCAGCAGTACGCACACA
Labelled probe	ACACTAGCCATCCTTACTGCGCTTCG

The RT-qPCR reactions were performed using the StepOnePlus Applied Biosystems machine. The program was set at: Reverse transcription (RT) 10 min at 50 °C; Inactivation 5 min at 95 °C; and then 40 cycles of denaturation 10 s at 95 °C and annealing/extension 30 s at 60 °C.

#### Infectious virus assessment by TCID_50_ assay in hamster lungs

All lung samples had detectable viral RNA and were subsequently examined for infectious virus by TCID_50_ assay. Briefly, appropriate amounts of tissues (less than 0.1 g) were homogenized in the TissueLyserII (6 min, 30 Hz) in DMEM media for a 1/10 tissue to media (w/v) dilution. The homogenate was centrifuged at 5000×*g* for 5 min. The supernatant was transferred to a clean tube for determination of TCID_50_ by cell culture. The supernatant was serially diluted (1:10) in DMEM with 2% FBS, 1X Penn/Strep. The assays were conducted in 96-well plates using Vero’76 cells (ATCC CRL-1587). Fifty microliters of the serially diluted tissue supernatant or nasal washes was added to 96-plate wells with pre-seeded Vero76 cells, in triplicate. The plates were incubated under 5% CO_2_ for 1 h at 37 ℃. Then the inoculum was removed and replaced with fresh complete DMEM containing 2% FBS (Sigma SAFC Lot 14G420), 1X Penn/Strep. The plates were incubated at 37 ℃, 5% CO_2_ for desired length of time. Then, the cytopathic effect (CPE) of cells in each well was examined under microscopes at 3 days post-infection. The experiment was performed in triplicate. Media alone and SARS-CoV-2 virus were used as negative or positive controls, respectively.

The virus was quantified and reported in TCID_50_/gram. Tissue viral loads were calculated using the Spearman-Karber method^58^. The lower limit of quantitation (LLOQ) was 6.32 × 10^1^ and the lower limit of detection (LLOD) was 1.36 × 10^1^ per sample.

### Histopathology

At 10 dpi, the harvested lungs were perfused with 10% neutral buffered formalin at room temperature and placed in a jar of formalin. After 7 days of formalin fixation in the BSL3 laboratory, the tissues were transferred to a container with fresh formalin and moved to a BSL2 laboratory. After 24 h the left lung lobes were submitted to a pathology facility for embedment, sectioning, and staining with haematoxylin and eosin stain (H&E) for light microscopic examination^59^. Slides were evaluated by a board-certified pathologist blinded to study groups using the scoring key described in Table 2 to evaluate inflammation of each type of tissue. To evaluate the lung pathology, individual scores for severity of inflammation, distribution of inflammation, extent of type II pneumocyte hyperplasia, and severity of hemorrhage in the lungs were assigned as described in Table 2 and an overall score was assigned based on the severity of these lesions altogether. Lung histopathological lesions were graded on a 4-point scale based on severity.

**Table 2:** Scoring matrix for lung pathology categories.

Category	Criteria	Scoring system
Inflammation	Intensity of neutrophilic infiltrate in affected areas	0: absent
		1: minimal
		2: mild
		3: moderate
		4: severe
% Distribution	Proportion of parenchyma affected	1: < 25%
		2: 26–50%
		3: 51–75%
		4: 76–100%
Type II pneumocyte hyperplasia	Extent of hypertrophy of alveolar pneumocytes	1: < 25%
		2: 26–50%
		3: 51–75%
		4: 76–100%
Hemorrhage	Intensity of hemorrhage in affected areas	0: absent
		1: minimal
		2: mild
		3: moderate
		4: severe

### Statistical analysis

All data analysis was conducted using ‘R’ (4.0.2) software. Statistical significance of the effect of COVID-HIGIV compared to PBS controls was determined using ANOVA (Analysis of Variance) followed by Dunnett’s pairwise comparison (virology data), nested ANOVA followed by Dunnett’s pairwise comparison (body weight data) and non-parametric Kruskal Wallis test followed by Dunn’s test (histopathology). Before implementing the parametric test, the normality of the data was tested using the Shapiro–Wilk test for normality. For all pairwise comparisons, p < 0.05 was considered significant. No animals were excluded from analysis. Data in the figures are presented for body weights as mean ± SEM or for peak body weight loss and tissue viral load as individual animals (circles), upper/lower quartiles (boxes), median (horizontal line) and ± 1.5 inter-quartile range (whiskers).

## Results

### Neutralization of SARS-COV-2 variants by COVID-HIG

To assess neutralizing potency against SARS-CoV-2 variants, two lots of COVID-HIGIV (pilot lot# PD_740_POC_17_001_006 and a representative clinical lot# 22002290) manufactured using convalescent plasma collected from donors prior to July 2020 were evaluated using wild-type neutralization assays against five SARS-CoV-2 variants (Alpha, Beta, Gamma, Delta and Omicron). The NT_50_ against each of the SARS-CoV-2 variants evaluated are given in Table 3.

**Table 3:** Neutralization titers for COVID-HIGIV lot #PD_740_POC_17_001_006 (manufactured in May 2020) and a representative clinical lot (lot# 22002290, manufactured in July 2020) against SARS-CoV-2 wild type and variant strains.

Variant	Neutralization titer	
	Pilot lot# PD_740_POC_17_001_006	Clinical lot# 22002290
Wild-type USA-WA1^1^	622	301
Alpha (B.1.1.7 CA)^2^	664	942
Beta (B.1.351)^3^	311	332
Gamma (P.1)^4^	1069	1054
Delta B.1.617.2^5^	193	188
Omicron BA.1^6^	106	152

^1^BEI Cat# NR 52,281, ^2^BEI Cat# NR-54011, ^3^BEI Cat# NR-54009, ^4^BEI Cat# NR-54984, ^5^BEI Cat #NR-55673, ^6^BEI Cat #NR-56461.

Both COVID-HIGIV lots were more effective at neutralizing the Alpha and Gamma variants than the wild-type WA1 isolate. Neutralizing titer for the Beta variant was comparable to the wild-type WA1 isolate but was reduced up to 3.2-fold or 5.9-fold for the Delta and Omicron variants, respectively. Overall, COVID-HIGIV effectively retains in vitro neutralizing potency against all SARS-CoV-2 variants tested to date, including the Delta and Omicron variants which have been shown to evade the neutralizing activity of most SARS-CoV-2 monoclonal antibody therapeutics^20^.

### COVID-HIGIV decreases clinical morbidity associated with SARS-Cov-2 infection in mice

**Figure 1:**
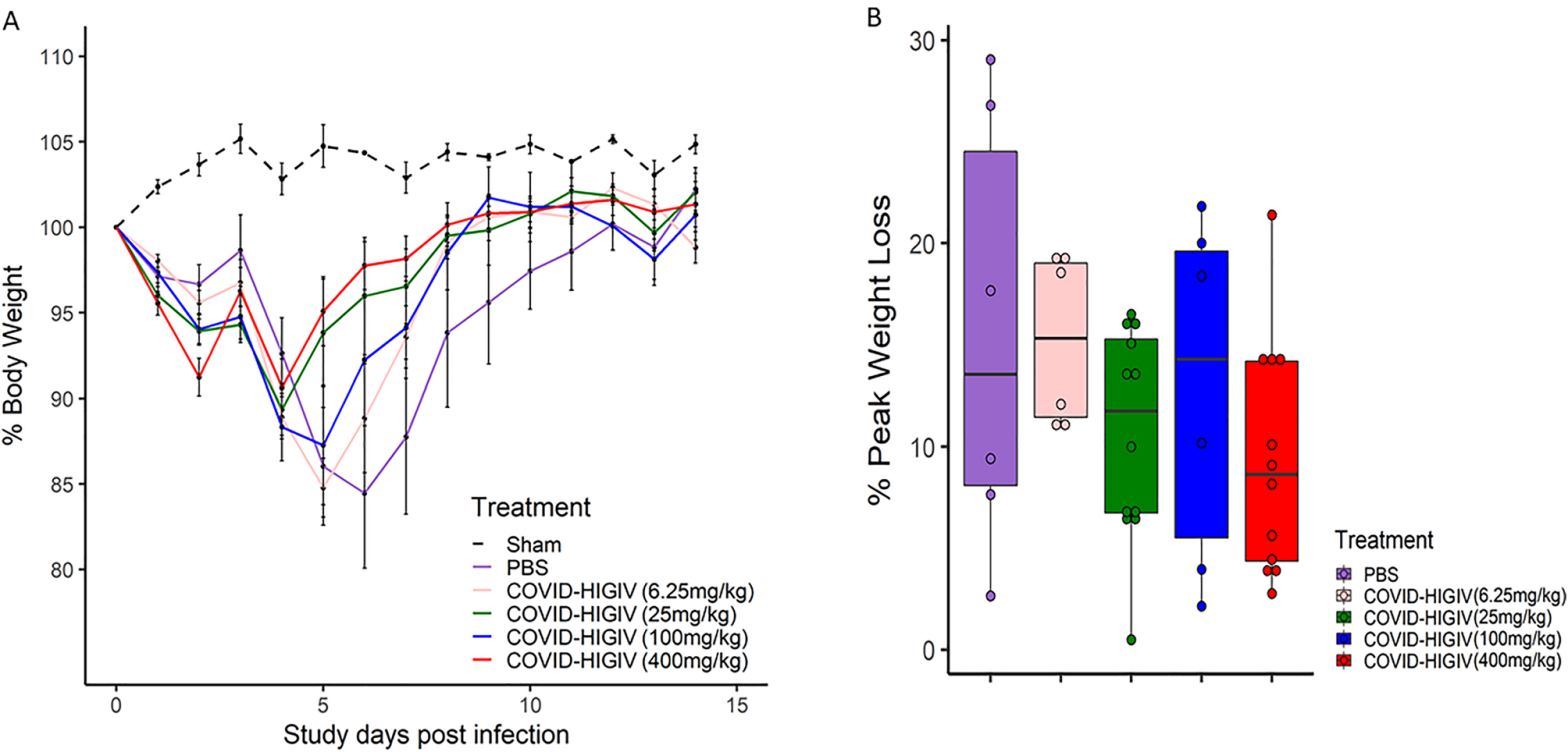
COVID-HIGIV treatment improves the clinical signs of SARS-CoV-2 infection in mice. Five groups of mice transduced with adenovirus carrying human ACE2 (N = 18) were challenged i.n. with SARS-CoV-2 (WA-1 strain), four groups were treated with different dosages of COVID-HIGIV (6.26, 25, 100 and 400 mg/kg dose) 6 h post infection, and the fifth group was PBS treatment control. An age matched sham infected group (N = 6) was also included as controls. Body weights for all surviving animals were collected each day until the end of the study. Figures represent (**A**) mean percent body weight, (**B**) mean percent peak body weight loss relative to the baseline weight on study day 0 before infection.

To measure the efficacy of COVID-HIGIV against the SARS-CoV-2 infection in terms of clinical morbidity, mice transduced with adenovirus expressing human ACE2 were infected with SARS-CoV-2 (strain WA-1) and then treated with COVID-HIGIV (400, 100, 25 and 6.25 mg/kg, i.p. route) 6 h post infection. Animals were observed daily for the body weight changes until the end of the study (14 dpi). For the body weight analysis, weights were normalized as a percentage of baseline body weight prior to statistical analysis. All mice, regardless of treatment, experienced similar levels of weight loss to 4 dpi. While animals in the PBS control group continued to lose substantial weight (15.5%) at 6 dpi, the animals in COVID-HIGIV treated groups started to recover by 4–5 dpi (Fig. 1A). Among COVID-HIGIV treated groups, a 400 mg/kg COVID-HIGIV dose showed lowest peak-body-weight loss of 9.4% followed by 25 mg/kg COVID-HIGIV (10.6%) and 100 mg/kg COVID-HIGIV (12.7%) groups. Both PBS (15.5%) and 6.25 mg/kg COVID-HIGIV (15.2%) groups had similar peak body weight loss. All groups recovered to baseline body weight within 14 dpi, suggesting a self-limiting COVID-like disease in this model. However, the recovery was faster in mice treated with COVID-HIGIV (all doses) compared to the PBS treated group (Fig. 1B).

### COVID-HIGIV decreases SARS-CoV-2 viral replication in the lungs of infected mice

**Figure 2:**
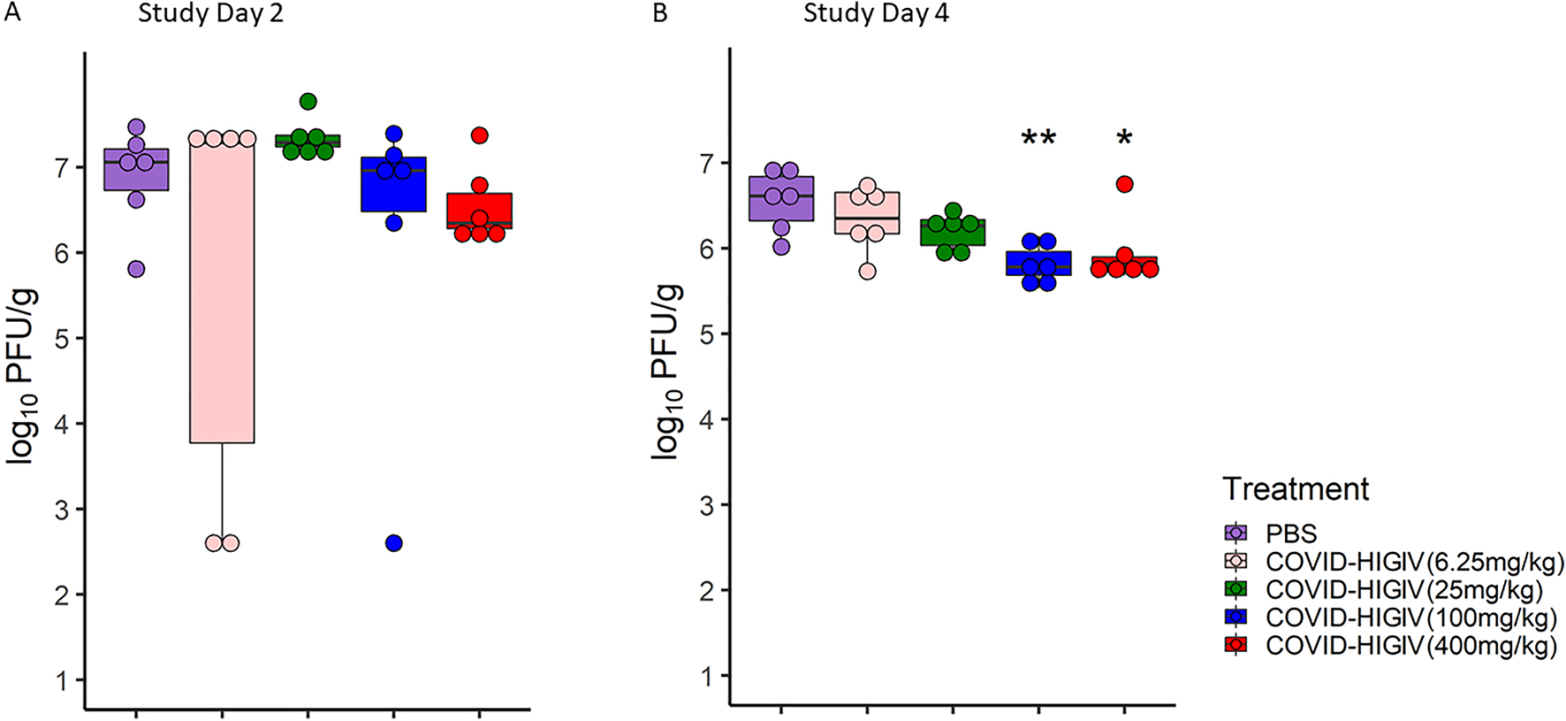
COVID-HIGIV treatment dose dependently reduced the live virus load in the mouse lungs. Five groups of mice transduced with adenovirus carrying human ACE2 (N = 18) were challenged i.n. with SARS-CoV-2 (WA-1 strain), four groups were treated with different dosages of COVID-HIGIV (6.26, 25, 100 and 400 mg/kg dose) 6 h post infection, and the fifth group was PBS treatment control. Six animals from each group were sacrificed on 2 and 4 dpi to harvest lung for ‘plaque forming unit assay’ for live virus load. Figures represent live virus load (**A**) 2 dpi, (**B**) 4 dpi. Statistical comparison was made using One-way ANOVA followed by Dunnett’s pairwise comparison with PBS treated group as control group. P values are reported as * < 0.05, ** < 0.01.

To determine how the COVID-HIGIV improved clinical outcome in terms of morbidity, virus replication was measured in the main target organ (lungs) of SARS-CoV-2 infected mice transduced with adenovirus expressing hACE2. In these mice, the lung viral loads were determined in tissues harvested at 2 and 4 dpi using plaque assay. These time points were selected based on available literature suggesting the peak virus load in lungs at 2 dpi, with virus replication subsiding by 5 dpi due to the self-limiting nature of SARS-CoV-2 infection in this model^35,50^. As expected, high virus titers (> 10^7^ PFU/g) were observed in control animals at 2 dpi and these titers were slightly reduced (~ 10^6.5^/g) by 4 dpi. A dose dependent reduction in viral load was observed in COVID-HIGIV treated animals at both 2 dpi and 4 dpi (Fig. 2A,B). At 4 dpi the lung viral load reduction was statistically significant in animals treated with 400 mg/kg (p < 0.05) and 100 mg/kg (p < 0.01) COVID-HIGIV compared to the PBS control group.

Thus, both high dose-levels (400 and 100 mg/kg) of COVID-HIGIV are effective against SARS-CoV-2 infection in this model as assessed by stringent live virus assays. Specifically, these results show that both dose levels reduced viral burden in a key target tissue (lungs) in mice at both critical time points of 2 and 4 dpi. In comparison to 400 and 100 mg/kg dose, the lower doses of COVID-HIGIV are less effective in reducing the viral burden in the lungs of infected mice.

### COVID-HIGIV decreases clinical morbidity associated with SARS-CoV-2 infection in hamsters

**Figure 3:**
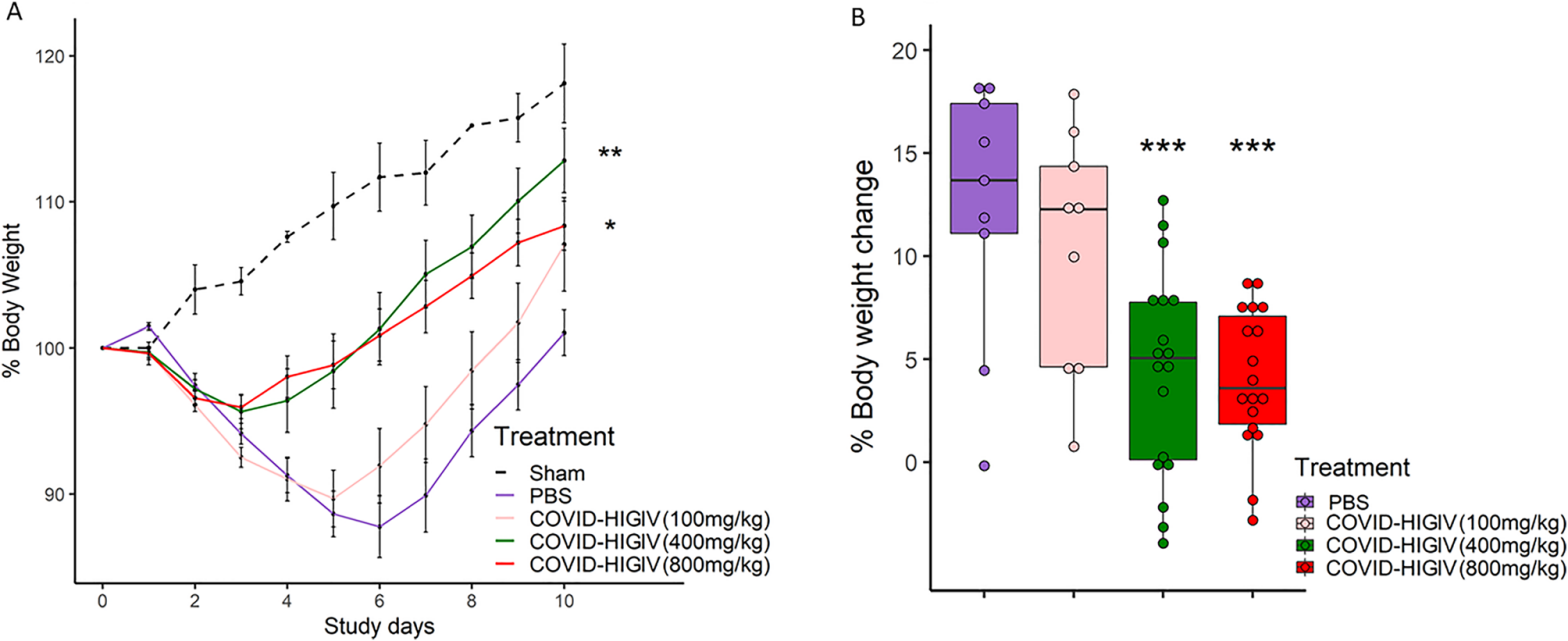
COVID-HIGIV treatment at dose ≥ 400 mg/kg significantly reduced clinical signs of SARS-CoV-2 infection in hamsters. Four groups of Hamsters (N = 18) were challenged i.n. with SARS-CoV-2 (1 × 10^5^ TCID_50_ in 100 mL; strain—SARS-CoV-2/Canada/ON/VIDO-01/2020), three groups were treated with different dosages of COVID-HIGIV (100, 400 and 800 mg/kg dose) 6 h before infection, and the fourth group was PBS treatment control. Body weights for all surviving animals were collected each day until the end of the study. Figures represent (**A**) mean percent body weight, (**B**) mean percent peak body weight loss relative to the baseline weight on study day 0 before infection. Statistical comparison was made using Nested One-way ANOVA followed by Dunnett’s pairwise comparison with PBS treated group as control group P values are reported as * < 0.05, ** < 0.01 and *** < 0.001.

To evaluate the efficacy of COVID-HIGIV against SARS-CoV-2 infection, wild-type golden Syrian hamsters were infected with SARS-CoV-2 (strain: Canada/ON/VIDO-01/2020) and treated prophylactically 6 h prior to infection with COVID-HIGIV (800, 400, 100 mg/kg, i.p. route). All hamsters except for sham infected (uninfected) animals exhibited transient body weight loss. A dose-dependent effect of COVID-HIGIV on clinical disease in terms of body weight loss was observed (Fig. 3A,B). Animals in the PBS control group continued to lose weight up to 12.4% on 6 dpi. Animals treated with 100 mg/kg COVID-HIGIV showed a similar degree of weight loss as PBS controls (10.3%) but began to recover at 5 dpi. Hamsters in the 400 and 800 mg/kg COVID-HIGIV treatment groups had significantly lower body weight loss compared to PBS controls (p < 0.001). Overall COVID-HIGIV 400 and 800 mg/kg significantly improved the overall magnitude and duration of body weight loss compared to PBS controls (p < 0.01 and p < 0.05, respectively). Overall, the effect of both 400 and 800 mg/kg dose of COVID-HIGIV on hamster body weight was comparable.

### COVID-HIGIV decreases viral replication in target tissue in hamsters

**Figure 4:**
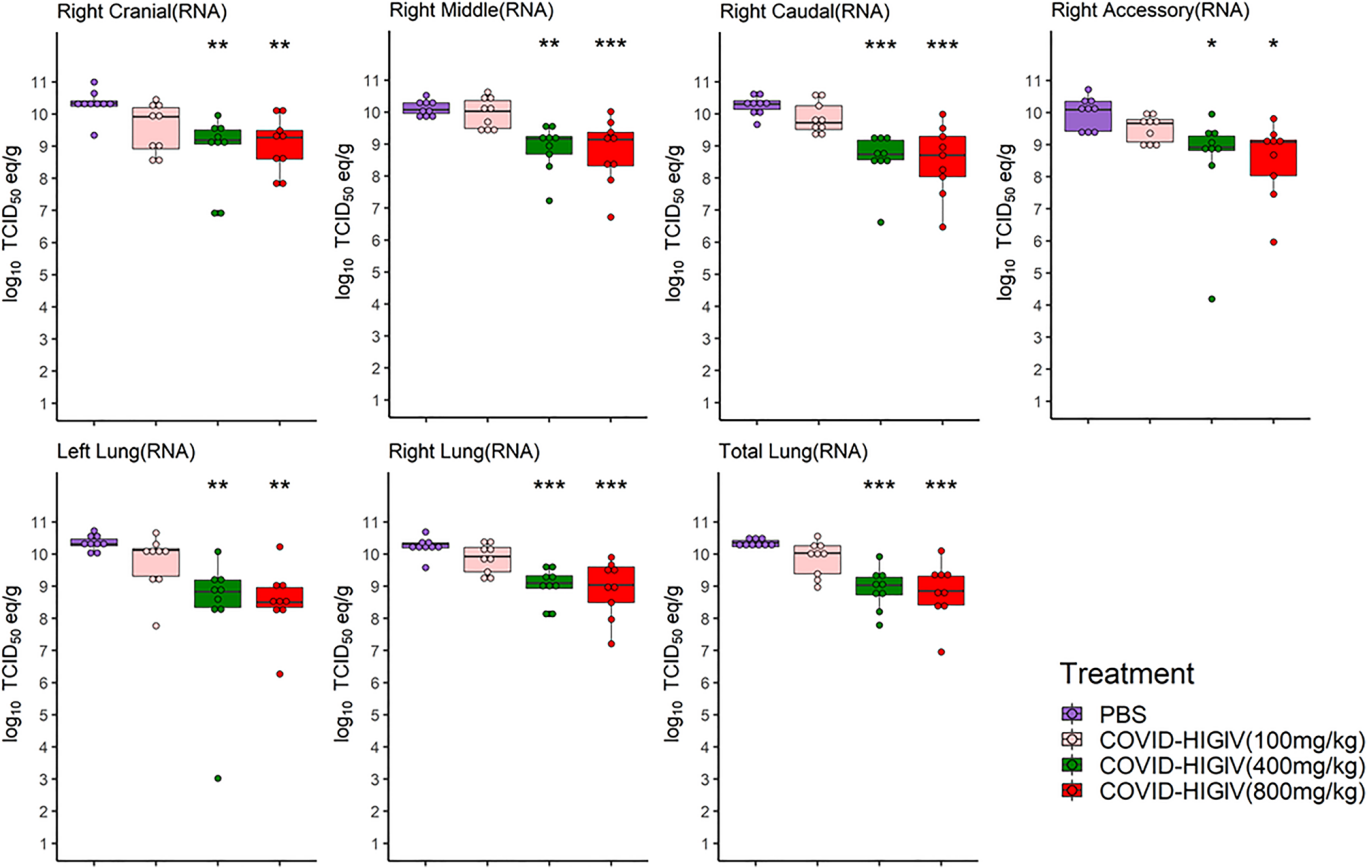
COVID-HIGIV treatment at dose ≥ 400 mg/kg significantly reduced SARS-CoV-2 virus sub-genomic RNA (sg-RNA) in hamster lungs. Four groups of Hamsters (N = 18) were challenged i.n. with SARS-CoV-2 (1 × 10^5^ TCID_50_ in 100 mL; strain—SARS-CoV-2/Canada/ON/VIDO-01/2020), three groups were treated with different dosages of COVID-HIGIV (100, 400 and 800 mg/kg dose) 6 h before infection, and the fourth group was PBS treatment control. Nine animals from each group were sacrificed at 3 dpi to harvest lung for sg-RNA load by RT-qPCR. Viral sg-RNA load was estimated in the four lobes of right lung (cranial, caudal middle and accessory lobes) and single lobe of the left lung. Viral load for entire right lung was extrapolated by the average viral loads for all four right lung lobes. Similarly, total lung viral load was extrapolated by the average viral loads for all four lobes from right lung and the single lobe from the left lung. Statistical comparison was made using One-way ANOVA followed by Dunnett’s pairwise comparison with PBS treated group as control group. P values are reported as ** < 0.01 and *** < 0.001.

To evaluate how COVID-HIGIV treatment improves morbidity in hamsters, viral burden in the lungs was assessed, a key target tissue for SARS-CoV-2 replication. In hamsters, lobe specific levels of viral sub-genomic RNA (sgRNA), and viable virus titers were assessed using RT-qPCR and TCID_50_ assay respectively, on 3 dpi coinciding with the expected peak viral load. Samples from all four lobes of right lung (cranial, caudal middle and accessory lobes) and the single lobe of the left lung were assessed. Viral loads for entire right lung were extrapolated by averaging the viral loads of all four right lung lobes. Similarly, total lung viral load was extrapolated by averaging the viral loads for all four lobes from the right lung and the single lobe from the left lung. Viral sgRNA and viable virus were uniformly distributed throughout the lungs of PBS control animals at 3 dpi. COVID-HIGIV treatment reduced viral sgRNA loads in the lungs in a dose-dependent manner (Fig. 4). A significant reduction in viral sgRNA was observed with 400 and 800 mg/kg dose of COVID-HIGIV compared to PBS controls (p < 0.01). Animals treated with 100 mg/kg COVID-HIGIV also showed a reduction in viral RNA in most lobes of the lung, however this reduction was not significant (Fig. 4). When data were pooled to assess viral load of total lung, both treatment with 400 and 800 mg/kg significantly reduced the lung viral RNA load (p < 0.001).

**Figure 5:**
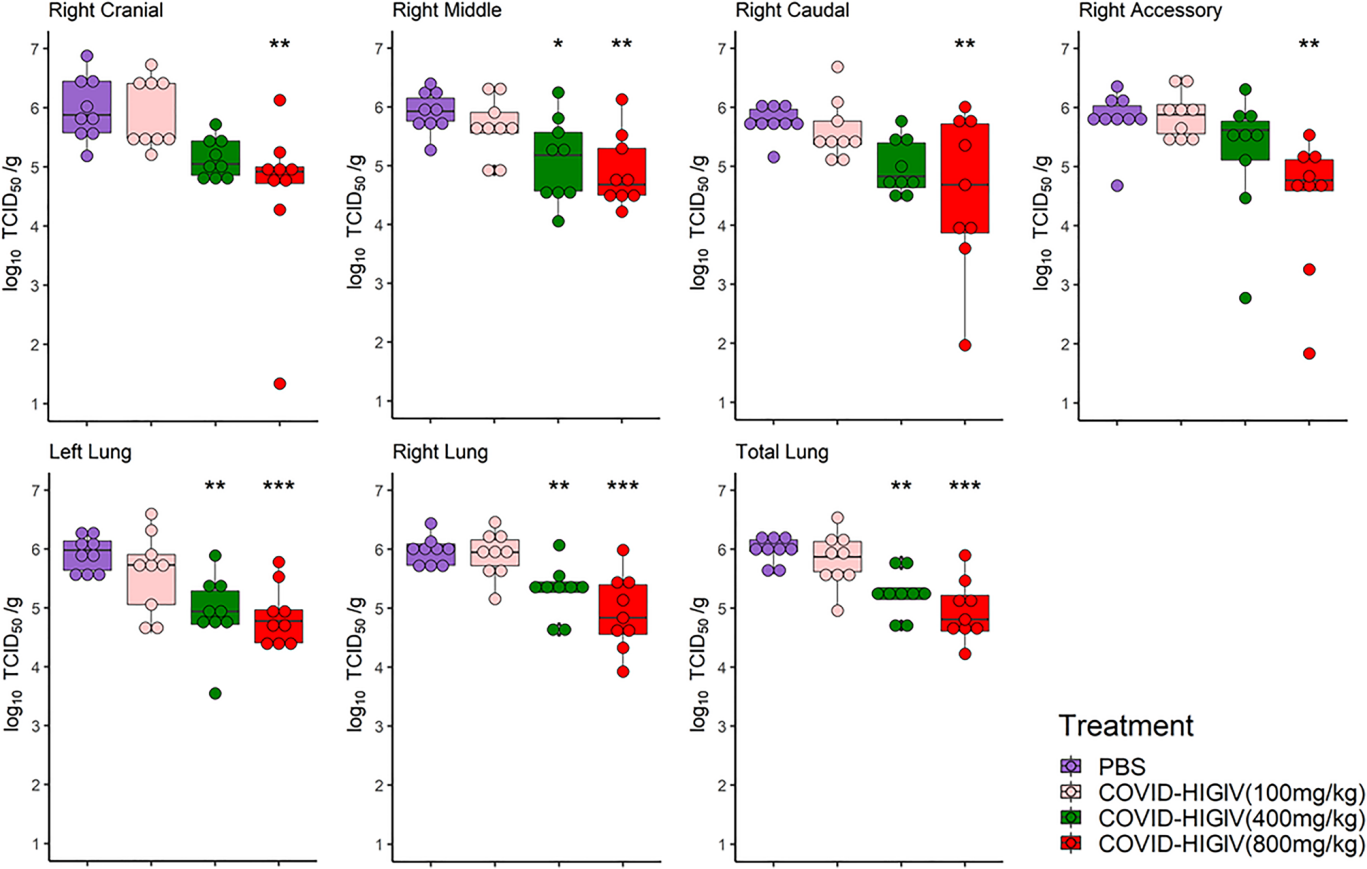
COVID-HIGIV treatment at dose ≥ 400 mg/kg significantly reduced SARS-CoV-2 live virus load in hamster lungs. Four groups of Hamsters (N = 18) were challenged i.n. with SARS-CoV-2 (1 × 10^5^ TCID_50_ in 100 mL; strain—SARS-CoV-2/Canada/ON/VIDO-01/2020), three groups were treated with different dosages of COVID-HIGIV (100, 400 and 800 mg/kg dose) 6 h before infection, and the fourth group was PBS treatment control. Nine animals from each group were sacrificed at 3 dpi to harvest lung for live virus load estimation by ‘tissue culture infection dose assay’. Live virus load was estimated in the four lobes of right lung (cranial, caudal middle and accessory lobes) and single lobe of the left lung. Viral load for entire right lung was extrapolated by the average viral loads for all four right lung lobes. Similarly, total lung viral load was extrapolated by the average viral loads for all four lobes from right lung and the single lobe from the left lung. Statistical comparison was made using One-way ANOVA followed by Dunnett’s pairwise comparison with PBS treated group as control group. P values are reported as * < 0.05, ** < 0.01 and *** < 0.001.

In line with the viral sgRNA results, a dose-dependent reduction in viable virus titers was observed in COVID-HIGIV treated groups (Fig. 5). COVID-HIGIV 800 mg/kg dose, significantly reduced the viable virus titers (p < 0.01) in all lung lobes compared to PBS control animals. COVID-HIGIV 400 mg/kg dose, also significantly reduced live virus titers (p < 0.05) in the right middle, left and overall right lung compared to the PBS control animals. Treatment with 100 mg/kg COVID-HIGIV dose group reduced viable virus titers in some lobes of the lung, but not significantly. Consistent with sgRNA load in total lung, viable virus titers were significantly reduced by treatment with 400 and 800 mg/kg COVID-HIGIV compared to the PBS control group (p < 0.01 and < 0.001 respectively).

### COVID-HIGIV is effective in reducing the lung pathology associated with SARS-CoV-2 infection

Severe lung pathology is observed in hamsters infected with SARS-CoV-2, similar to what is observed in human disease^39,56,57^. As the major consequences of SARS-CoV-2 infection are related to lung infection and associated lung pathology, the lungs of COVID-HIGIV treated and control hamsters were compared for infection related lung pathology.

**Figure 6:**
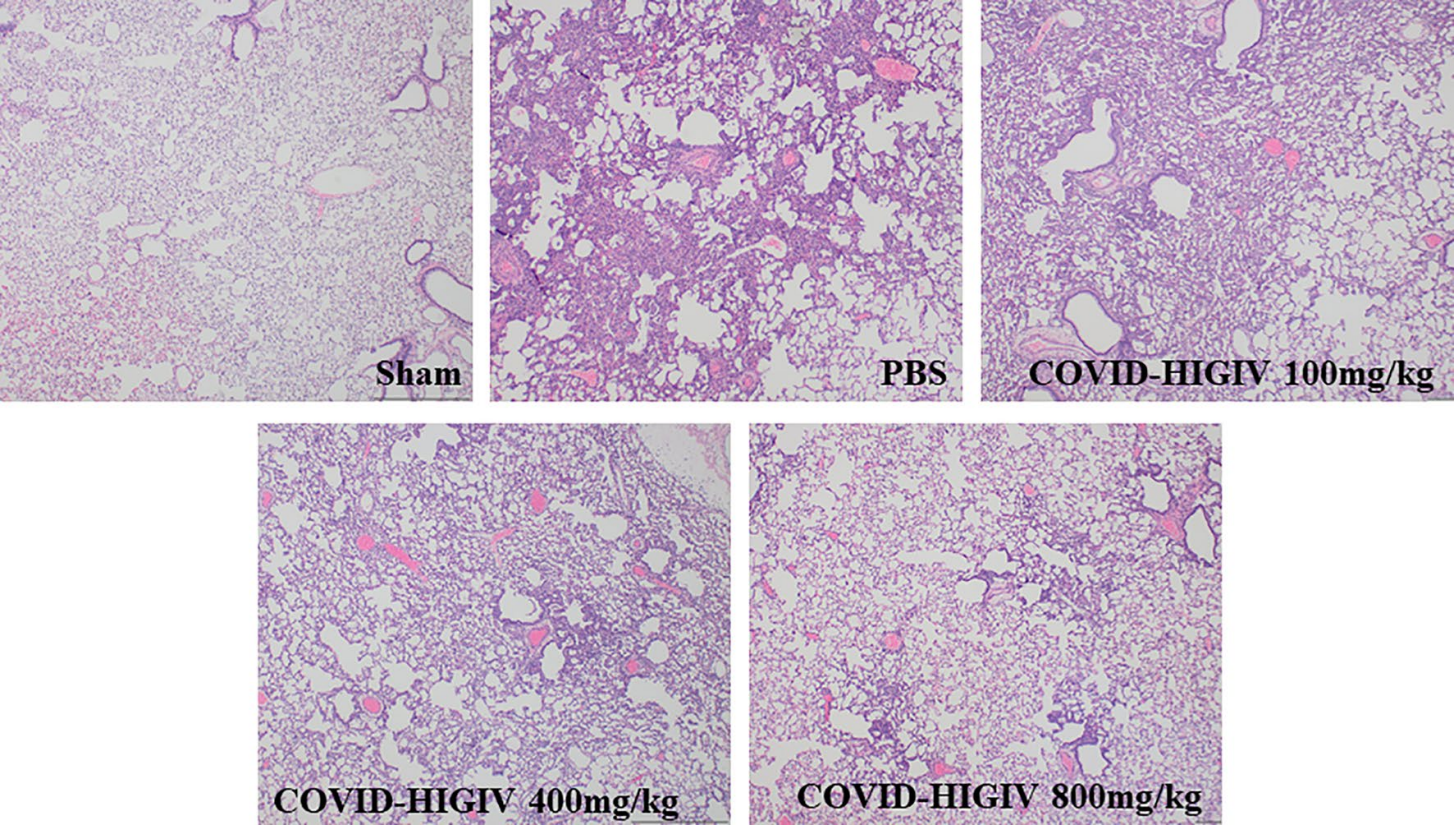
COVID-HIGIV treatment at dose ≥ 400 mg/kg significantly reduced SARS-CoV-2 infection induced lung pathology in hamsters. Four groups of Hamsters (N = 18) were challenged i.n. with SARS-CoV-2 (1 × 10^5^ TCID_50_ in 100 mL; strain—SARS-CoV-2/Canada/ON/VIDO-01/2020), three groups were treated with different dosages of COVID-HIGIV (100, 400 and 800 mg/kg dose) 6 h before infection, and the fourth group was PBS treatment control. Nine animals from each group were sacrificed at 10 dpi to harvest left lung for histopathology. Left lobe of the lung was fixed in paraformaldehyde and embedded in wax before sectioning at 5 µm thickness and staining with H&E for light microscopic examination at ×4  magnification. Pathology scoring was performed by the pathologist in a blinded manner.

PBS treated hamsters were characterized by inflammation and type II pneumocyte hypertrophy. COVID-HIGIV treatment reduced this pathology as well as the proportion of lung parenchyma affected by inflammation in a dose-dependent manner (Fig. 6, Table 4). Treatment with 400 or 800 mg/kg COVID-HIGIV significantly reduced inflammation severity (p < 0.05 and p < 0.01, respectively) and pneumocyte hypertrophy (p < 0.05 and p < 0.001 respectively). The percentage of inflamed lung parenchyma was also reduced at these doses but not significantly. The 100 mg/kg COVID-HIGIV dose also improved lung pathology compared to PBS controls, in particular inflammation severity and pneumocyte hypertrophy, however this improvement was not statistically significant. The overall pathologist score indicates that animals treated with ≥ 400 mg/kg COVID-HIGIV had healthier lungs compared to the PBS control group, but this improvement was only statistically significant for the 800 mg/kg COVID-HIGIV dose group (p < 0.01). While there was no improvement in overall pathology score for the 100 mg/kg COVID-HIGIV dose group compared to the PBS control group, also, there was no indication of enhanced pathology of disease at any dose evaluated. These results are consistent with the lung pathology of SARS-CoV2 infection in this model and show that COVID-HIGIV treatment reduces SARS-COV-2 induced lung pathology.

**Table 4:** Summary of lung histopathology findings.

Lesion type	Treatment	Histopathological score			
		Mode	Mean	N	P value
Inflammation severity	Sham	0	0	2	0.004
	PBS	3	2.78	9	–
	COVID-HIGIV 100 mg/kg	2	2.11	9	0.458
	COVID-HIGIV 400 mg/kg	2	1.67	9	0.041
	COVID-HIGIV 800 mg/kg	1	1.33	9	0.004
Affected parenchyma	Sham	0	0	2	0.003
	PBS	2	1.89	9	–
	COVID-HIGIV 100 mg/kg	1	1.78	9	1
	COVID-HIGIV 400 mg/kg	1	1.56	9	0.285
	COVID-HIGIV 800 mg/kg	1	1	9	0.01
Type-II pneumocyte hypertrophy	Sham	0	0	2	0.009
	PBS	1	1.11	9	–
	COVID-HIGIV 100 mg/kg	1	0.67	9	0.105
	COVID-HIGIV 400 mg/kg	1	0.56	9	0.038
	COVID-HIGIV 800 mg/kg	0	0.22	9	< 0.001
Overall pathology	Sham	0	0	2	0.002
	PBS	2	2.22	9	–
	COVID-HIGIV 100 mg/kg	2	1.78	9	0.433
	COVID-HIGIV 400 mg/kg	1	1.44	9	0.096
	COVID-HIGIV 800 mg/kg	1	1.11	9	0.007

## Discussion

Passive immunotherapy with plasma or immunoglobulin obtained from convalescent individuals recovered from COVID-19 and containing high neutralizing antibodies against SARS-CoV-2 is a promising therapy against COVID-19 where vaccines are either not available or exempted for medical/non-medical reasons. Additionally, passive immunotherapy could be useful in a prophylactic setting for at-risk populations. Although the efficacy of convalescent plasma against the progression of COVID-19 disease is yet to be proven in randomized clinical trials^60^, the recent meta-analysis suggests that the convalescent plasma is not an effective therapy^31^. This is likely due to treatment time and the IgG titer in plasma against the SARS-CoV-2 spike protein. These studies suggest that early administration of high titer convalescent plasma could reduce the progression of the disease^61,62^.

This study describes the use of COVID-HIGIV as an effective countermeasure against SARS-CoV-2 infection in mice and hamsters. We evaluated in detail, the in vivo efficacy of COVID-HIGIV in both Syrian golden hamsters and mice transduced with adenovirus carrying hACE2. The SARS-CoV-2 virus replicates very efficiently in both mice transduced with adenovirus carrying hACE2 and hamsters resulting in a disease characterized by weight loss and high lung viral load. In hamsters SARS-CoV-2 infection also caused distinct lung pathology. COVID-HIGIV was effective in mice against morbidity by decreasing virus replication in the lungs. Similarly, COVID-HIGIV effectively reduced the clinical disease by reducing lung viral burden and SARS-CoV-2 induced lung pathology in hamsters. These findings support further development of COVID-HIGIV as a candidate for the treatment and/or post-exposure prophylaxis of COVID-19 patients.

Currently, there are four vaccines either licensed or approved under Emergency Use Authorization (EUA)^6^ and there are several mAb therapeutics approved under EUA^14–17^ for the treatment of COVID-19. There are many more mAbs reported to be efficacious in animal models^35,36,38,63^; however, the emergence of variant strains has put many monoclonal therapies at the risk of losing efficacy^64–66^. The omicron variant has become dominant SARS-CoV-2 in the United States and has markedly reduced in vitro susceptibility to several SARS-CoV-2 mAbs, including bamlanivimab plus etesevimab and REGEN-COV^20^.

Polyclonal antibody therapy such as COVID-HIGIV has the potential to provide an additional line of defence to prevent disease progression or complications^67^ and due to its polyclonal nature, it is less susceptible to antibody escape as a result of mutations present in variants of SARS-CoV-2^68,69^. The ability of COVID-HIGIV to cross-neutralize SARS-CoV-2 variants highlights the potential of this product to be used against variants.

In the mouse model, PBS control animals showed robust lung viral load and body weight loss, in line with published reports^35,49^. COVID-HIGIV reduced levels of viable virus in lung tissue in a dose dependent manner on 2 and 4 dpi compared to PBS controls. These reductions were statistically significant for both the 400 and 100 mg/kg COVID-HIGIV dose groups where viral load was reduced by ~ 1 log PFU/g of tissue. COVID-HIGIV also reduced the clinical severity of SARS-CoV-2 infection as demonstrated by substantial reduction in body weight loss and faster recovery.

In hamsters, lung viral load and body weight loss in PBS control group were comparable to already reported data in the literature^39,57^. COVID-HIGIV treatment was effective in reducing viral sgRNA and live virus in a dose-dependent manner. This reduction of viral load was statistically significant for the 400 and 800 mg/kg dose of COVID-HIGIV, where a > 1 log reduction in the viral load (sgRNA and viable virus) was observed. This reduction in viral load correlated with a significant reduction of infection associated lung pathology and clinical morbidity in the form of reduced magnitude and duration of body weight loss.

Both mouse and hamster models have been used to evaluate the efficacy of mAbs. Mice transduced with adenovirus carrying human ACE2 have been used to demonstrate benefit of mAb 1B07 when administered 24 h prior to infection. This antibody effectively reduced lung virus load and prevented body weight loss significantly^35^ when given prophylactically before infection. This report demonstrates the effect of human hyperimmunes specific for COVID-19 in mice transduced with adenovirus carrying human ACE2. Similarly, the hamster model has been established for evaluation of vaccines and therapeutics against SARS-CoV-2 infection. In this model, mAbs have been shown to reduce the live virus load, lung pathology and the clinical signs of disease^37,38^. Additionally, there are a few reports that have evaluated the efficacy of hamster convalescent serum and human hyperimmune IgG product in the hamster model of SARS-CoV-2^39,70^. Hamster convalescent serum significantly reduced the lung live virus load by ~ 3 and ~ 2 log_10_ PFU/g, when administered 1 and 2 days after infection, respectively^39^. It is difficult to compare results from this study head-to-head with the published work on mAbs and hamster convalescent plasma due to variation in study design, differences in potency and dose of the product. However, the results presented here indicate that COVID-HIGIV provides protection against lung viral loads similar to published reports on mAbs and hamster convalescent plasma. This protection was obtained despite lower circulating neutralizing antibody titer (estimated based on total administered titer adjusted for hamster plasma volume), compared to the circulating neutralization titers of mAbs and hamster plasma used in other published reports^35,38,39,70^.

A recently published work showed that a human hyperimmune IgG product administered 2 days after SARS-CoV-2 infection in hamsters via the i.v. route reduced the sgRNA^70^. Although the viral load reduction was significant, the magnitude of reduction was < 1 log. This study also showed a positive impact of human hyperimmune on body weight loss at day 6 and 7 post-infection^70^. The overall impact of human hyperimmune IgG product in this study was inferior to COVID-HIGIV suggesting that delayed treatment with human hyperimmune IgG may not be as effective as early intervention in hamster model. The hamster model is considered a more severe disease model for COVID-19 than the non-human primate model. Considering the pharmacokinetic limitations of the antibodies administered via the i.p route combined with rapid replication of SARS-CoV-2, it has been shown that the treatment scenario in the model is not practical^70^. However, this is a model-specific issue, and COVID-HIGIV could still benefit humans when given intravenously post-exposure prophylaxis or at early stages of the disease. It is important to note that, despite a statistically significant reduction in the lung viral loads in COVID-HIGIV treated animals, there was still a substantial amount of infectious virus in the lungs of both mice (~ 10^6^ PFU/g) and hamsters (~ 10^5^ TCID_50_/g) on all outcome days. This could be due to an insufficient amount of antibody penetrating the lung compartment, consistent with the studies suggesting about 10–15% of the circulating antibodies reach lungs^71–73^. This suggests a localized delivery of antibodies to the lungs via intranasal or inhaled route of delivery could be more effective in neutralizing SARS-CoV-2 in the lungs, and studies have already begun to evaluate the efficacy of inhaled antibodies against SARS-CoV-2 infection^63,74^.

Despite the limitations, the observed efficacy of COVID-HIGIV treatment, when administered 6 h before infection in hamsters or 6 h after infection in mice transduced with adenovirus carrying human ACE2, represent an early treatment scenario. The hamster model of SARS-CoV-2 infection represents some of the features of severe human infections, and SARS-CoV-2 infection in mice transduced with adenovirus carrying hACE2 represents milder disease^50,57,75–77^. These studies demonstrate that COVID-HIGIV is effective in treating mild to moderate and severe SARS-CoV-2 infection and suggest that COVID-HIGIV could significantly inhibit the progression of COVID-19 when administered as early as possible to at risk populations.

## Data Availability

All data generated or analysed during this study are included in this published article. COVID-HIGIV product may be requested from the corresponding author for independent evaluation.
